# Does pragmatically structured outpatient dietary counselling reduce sodium intake in hypertensive patients? Study protocol for a randomized controlled trial

**DOI:** 10.1186/s13063-015-0794-y

**Published:** 2015-06-17

**Authors:** Marcel Ruzicka, Tim Ramsay, Ann Bugeja, Cedric Edwards, George Fodor, Anne Kirby, Peter Magner, Brendan McCormick, Gigi van der Hoef, Jessica Wagner, Swapnil Hiremath

**Affiliations:** Division of Nephrology, The Ottawa Hospital and the University of Ottawa, 5-11 1967 Riverside Drive, Ottawa, ON K1H7W9 Canada; Kidney Research Centre, The Ottawa Hospital, 1967 Riverside Drive, Ottawa, ON K1H7W9 Canada; Prevention and Rehabilitation Centre, University of Heart Institute, 40 Ruskin Street, Ottawa, ON K1Y4W7 Canada; Clinical Epidemiology Program, Ottawa Hospital Research Institute, 501 Smyth Road, Ottawa, ON K1H8L6 Canada

**Keywords:** Hypertension, Blood pressure, Salt, Sodium, Dietary modification, Dietary counselling, Lifestyle modification, Clinical trial

## Abstract

**Background:**

Hypertension is highly prevalent among adults, and is the most important modifiable risk factor for cardiovascular events, in particular stroke. Decreasing sodium intake has the potential to prevent or delay the development of hypertension and improve blood pressure control, independently of blood pressure lowering drugs, among hypertensive patients. Despite guidelines recommending a low sodium diet, especially for hypertensive individuals, sodium intake remains higher than recommended. A recent systematic review indicated that the efficacious counselling methods described in published trials are not suitable for hypertension management by primary care providers in Canada in the present form. The primary reason for the lack of feasibility is that interventions for sodium restriction in these trials was not limited to counselling, but included provision of food, prepared meals, or intensive inpatient training sessions.

**Methods/design:**

This is a parallel, randomized, controlled, open-label trial with blinded endpoints. Inclusion criteria are adult patients with hypertension with high dietary sodium intake (defined as ≥100 mmol/day). The control arm will receive usual care, and the intervention arm will receive usual care and an additional structured counselling session by a registered dietitian, with four follow-up telephone support sessions over four weeks. The two primary outcomes are change in sodium intake from baseline, as measured by a change in 24-hour urinary sodium measurements at four weeks and one year. Secondary outcomes include change in blood pressure (as measured by 24-hour ambulatory monitoring), change in 24-hour urinary potassium, and change in body weight at the same time points.

**Discussion:**

Though decreasing sodium intake has been reported to be efficacious in lowering blood pressure, there exists a gap in the evidence for an effective intervention that could be easily translated into clinical practice. If successful, our intervention would be suitable for outpatient programs such as hypertension clinics or interprofessional family practices (family health teams). A negative, or partially negative (positive effect at four weeks with attrition by 12 months) trial outcome also has significant implications for healthcare delivery and use of resources.

**Trial registration:**

The trial was registered with Clinicaltrials.gov (identifier: NCT02283697) on 2 November 2014.

**Electronic supplementary material:**

The online version of this article (doi:10.1186/s13063-015-0794-y) contains supplementary material, which is available to authorized users.

## Background

Hypertension is highly prevalent among adult Canadians (about 20 % in those aged 20 to 79 years), and is the most important modifiable risk factor for cardiovascular events, in particular stroke [1, 2]. The relationship between sodium intake as assessed from 24-hour urinary sodium and age-related increase in systolic blood pressure (BP) has been well established [3]. Furthermore, decreasing sodium intake has the potential to prevent or delay the development of hypertension and improve BP control, independently of BP lowering drugs, among hypertensive patients [4, 5]. With this in mind, it is not surprising that the restriction of dietary salt intake for the prevention and management of hypertension is a strongly endorsed health measure [6–8]. However, Health Canada survey findings indicate that the current average salt intake among Canadian adult males and females ranges between 6.5 and 9 g (or between 2,600 and 3,600 mg of sodium)/day, with men consuming more dietary salt than women [9]. However, according to the 2014 Canadian Hypertension Education Program (CHEP) guidelines, sodium intake should not exceed 2,000 mg/day [6]. Indeed, an individual would have to reduce their sodium intake by 50 to 70 %, depending on their hypertensive status, to reach the CHEP recommended target [6].

This magnitude of salt intake reduction to levels envisaged by the CHEP is projected to have a significant impact on BP level and on adverse vascular outcomes of hypertension [10]. It is estimated that 1.65 million deaths worldwide were due to cardiovascular causes from a daily sodium consumption of more than 2 g [11]. It is also estimated that the associated healthcare cost savings would amount to 10 to 24 billion US dollars annually [10]. Given the proven relationship between the dietary sodium intake and hypertension, the known risk of adverse outcomes of hypertension, and the individual and public health financial, health, and social benefits of low salt intake, it is somewhat surprising that salt intake remains stagnantly high over the last several decades, despite many public health efforts [9, 12].

Canadian adults are mostly diagnosed with high BP during routine annual check-ups with their family physician. Family physicians are expected to establish the diagnosis and to initiate hypertension therapy. Since the majority of hypertensive patients in Canada have mild and uncomplicated hypertension, primary care physicians also continue to manage a significant proportion of patients with hypertension [13]. Thus, with regards to the treatment of hypertension, family physicians are really the ‘front-liners’, who introduce both lifestyle modifications and pharmacological interventions to individual patients and make further adjustments in therapy to achieve target BP. The CHEP provides detailed guidelines on the diagnosis and treatment of hypertension to Canadian family physicians [6]. These guidelines include specific algorithms for the diagnosis and management of hypertension [6]. In the setting of very busy primary care practices, these algorithms are crucial to facilitate making the diagnosis, initiating and maintaining hypertension therapy, and ensuring that patients are engaged and informed.

However, in contrast to the specific algorithms provided for the diagnosis and pharmacological treatment of hypertension, algorithms on how to achieve the rigorous targets for dietary sodium intake are absent [6]. CHEP specifically endorses the Dietary Approaches to Stop Hypertension (DASH) diet [4] because it is well described, reproducible, and leads to sustainable decreases in salt intake. However, what is not clear from the guidelines is details about the most efficacious method of counselling on dietary sodium restriction to achieve potentially very rigorous targets, how to assess compliance and progress of an individual patient with regards to sodium intake restriction, and finally how to include these potentially time-consuming tasks into the daily routine of busy Canadian family physicians. Indeed, lack of time and inadequate compensations have been reported as the strongest barriers to providing nutrition guidance by US and Canadian primary practices [14–16]. These results should not come as surprise once one appreciates how much time primary care physicians in North America have ‘available’ for their patients. For example, results of the National Review of Medicine 2006 Practice Management Survey showed that majority of family physicians in Canada see 26 to 50 patients per day [17]. Further, 42 % of 700 responders reported an increase in patient load in the last 12 months [17]. A recent systematic review indicated that there are well-described, and therefore, easy to reproduce counselling methods on efficacious low sodium diets [18]. However, none of these counselling methods are suitable for hypertension management by primary care providers in Canada in the present form. The primary reason for the lack of feasibility in the outpatient settings in Canada is that interventions for salt restriction in these trials was not limited to mere counselling, but included provision of food, prepared meals, or intensive inpatient training sessions [18].

Hence, given that data from clinical trials support the efficacy of dietary sodium reduction in lowering BP, but with a paucity of research into clinically feasible and logistically simple methods of implementing this, we have designed a pragmatic clinical trial to test if the addition of lead counselling by a registered dietitian, in comparison to usual care, results in a change in dietary sodium intake.

## Methods/design

### Study design

The study is designed as a pragmatic, parallel, open-label, randomized controlled trial with blinded endpoints. Since the intervention is a counselling session, blinding will not be possible. However, the primary outcome of the study is a change in urinary sodium, which is an objective endpoint, and the laboratory personnel performing the measurement and the research personnel analyzing the measurements will be blinded to treatment assignment. The randomization process will consist of a computer-generated random listing of the treatment allocations in variable permuted blocks, with concealment of allocation using sealed envelopes. The Standard Protocol Items: Recommendations for Interventional Trials (SPIRIT) checklist and the World Health Organization (WHO) Trial Registration Data Set Checklist are also presented (see Additional file 1 and Table 1).

**Table 1:** World Health Organization Trial Registration Data Set Checklist

Data category	Information
Primary registry and trial identifying number	ClinicalTrials.gov: NCT02283697
Date of registration in primary registry	4 November 2014
Secondary identifying numbers	-
Source(s) of monetary or material support	The Ottawa Hospital Academic Medical Organisation
Primary sponsor	The Ottawa Hospital Academic Medical Organisation
Secondary sponsor(s)	Ottawa Hospital Research Institute
Contact for public queries	Gigi van den Hoef +16137985555 ext 82514
Contact for scientific queries	Marcel Ruzicka, MD PhD
	Swapnil Hiremath, MD MPH
Public title	Sodium counselling in hypertension
Scientific title	Does Pragmatically Structured Outpatient Dietary Counselling Reduce Sodium Intake in Hypertensive Patients?
Countries of recruitment	Canada
Health condition(s) or problem(s) studied	Hypertension
Intervention(s)	Active comparator: standard of care + Dietitian lead counselling
	Control comparator: standard of care
Key inclusion and exclusion criteria	Inclusion criteria:
	- adult patients (>18 years) with
	- hypertension defined as daytime blood pressure (BP) readings above 140/90 mmHg (as assessed from 24-hour ambulatory BP monitoring (ABPM)) without treatment
	- and/or any patient with treated hypertension irrespective of BP load based on 24-hour ABPM.
	Exclusion criteria:
	- Pregnant patients (since pregnancy and hypertension requires different dietary advice)
	Patients with following conditions:
	- eGFR <45 ml/min/1.75 m^2^,
	- active infection (defined as being on active anti-microbial treatment),
	- recent acute coronary syndrome (myocardial infarction or revascularisation) within 6 months,
	- psychiatric disorders and/or otherwise unable to sign consent,
	- patients with clinically manifested generalized and/or cardiac volume overload (including ascites and congestive heart failure) who may require immediate changes in diuretic therapy (at the discretion of treating hypertension specialist).
	- Inability to provide informed consent
Study type	Interventional
	Allocation: randomized
	Intervention model: parallel assignment
	Masking: blinding of outcomes assessor only
	Primary purpose: treatment
Date of first enrolment	June 2015 (anticipated)
Target sample size	120
Recruitment status	Funded, approved, enrolment to begin soon
Primary outcome(s)	Change in 24-hour urinary sodium from baseline at 4 weeks and 12 months
Key secondary outcomes	Changes in 24-hour urinary potassium, body weight, and BP

### Setting

The trial will be conducted at a tertiary care academic health sciences centre in Canada. The setting is a Renal Hypertension Clinic, which receives consultations for difficult-to-control hypertension. Potential participants will be identified from the clinic and approached by a member of the health care team to ascertain their willingness to be contacted by the research personnel. Once the patient has agreed to be contacted by the research personnel, a member of the research team will contact the patient and will briefly describe the trial and determine if the patient is interested in participating. If so, a copy of the consent form will be provided to the patient.

After reading the consent form, if the patient expresses interest, they will be asked to attend a screening visit where a study team member will explain the protocol, and potential risks and benefits again in detail. If the patient chooses to enroll in the study, the study coordinator will obtain written informed consent and ensure all necessary information is obtained. The research coordinator will ensure that, during the consent process, there is no overture of coercion, duress, or undue incentive.

### Study population

The inclusion criteria for this study are as follows:

1. Adult patients (>18 years);
2. Patients with hypertension defined as either daytime BP readings above 140/90 mmHg (as assessed from 24-hour ambulatory BP monitoring (ABPM)) without treatment, or any patient with treated hypertension irrespective of BP load; and
3. Dietary sodium intake >100 mmol/day, as measured with 24-hour urinary collection.

The exclusion criteria for this study are as follows:

1. Pregnant patients (since pregnancy and hypertension requires different dietary advice);
2. Patients with an estimated glomerular filtration rate (eGFR, measured using the MDRD formula [19]) <45 ml/min/1.75 m^2^;
3. Patients with an active infection (defined as being on active anti-microbial treatment);
4. Patients with recent acute coronary syndrome (myocardial infarction or revascularisation) within the last six months;
5. Patients with psychiatric disorders and/or who are otherwise unable to sign consent; and
6. Patients with clinically manifested generalized and/or cardiac volume overload (including ascites and congestive heart failure), who may require immediate changes in diuretic therapy (at the discretion of treating hypertension specialist).

### Interventions

Patients randomized to the control arm will receive a standard endorsement of low salt diet and other non-pharmacological interventions, such as moderation of alcohol intake, optimal body weight, and daily exercise, from a hypertension nurse and physician, which is the standard of care.

Patients randomized to the intervention arm will receive usual care and an additional one-on-one (family members allowed) one-hour long counselling session from a registered dietitian. The dietitian will assess the patient’s eating habits and identify factors involved in high sodium intake, focusing on portion size, processed food, snacking habits, eating out, and reading labels. Apart from counselling tailored to the cause and source of high sodium, a handbook with advice on decreasing sodium in diet will be provided. This will be followed by four weekly, 30-minute long follow-up sessions by telephone to provide support, address compliance, and answer any questions raised by patients and family members.

### Measurements

We will evaluate dietary sodium intake by measurement of 24-hour urinary sodium before and after counselling. We will not allow changes in diuretic therapy within a week prior to counselling and throughout the four weeks of the trial duration, and patients in whom an urgent change in diuretic therapy is anticipated will be excluded from the trial. This approach will ensure that baseline 24-hour urinary sodium reflects steady state and represents 24-hour dietary sodium intake. Similarly, changes in 24-hour urinary sodium four weeks post-intervention will reflect changes in patients’ dietary sodium intake with no interference of the effect of diuretics. In studies using the DASH diet and longer follow-up periods, the effect of intervention is attenuated once the food is not provided to the patient, and so does the effect on BP [20, 21]. Hence, we will have the sodium intake re-assessed 12 months post-intervention to assess for the long term effectiveness of this method.

**Fig. 1:**
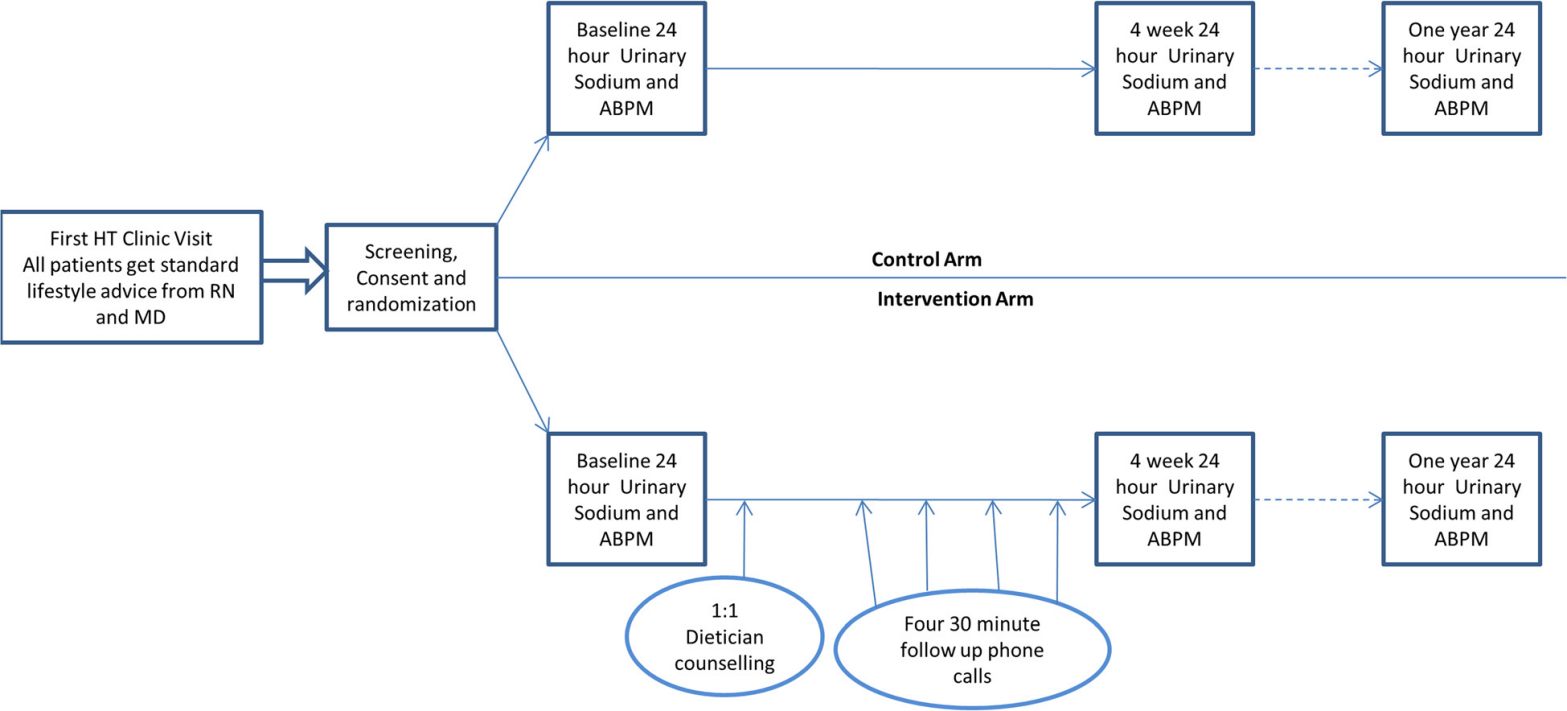
Study flow diagram showing time points at which data will be collected. Figure legend: HT: Hypertension; MD: Medical Doctor, physician; RN: Registered Nurse; ABPM: Ambulatory Blood Pressure Monitoring

24-hour ABPM will be done before and after dietary counselling within 24 to 48 hours of 24-hour urinary collection for sodium. This will allow us to correlate any changes in dietary sodium intake with changes in overall BP load. In addition, we will also measure changes in 24-hour urinary potassium, and changes in body weight. Lastly, a simple one-page survey will be administered to all patients to assess their perceptions of potential barriers to dietary sodium reduction at the end of the 12-month follow-up period. The time points of the intervention and data collection are shown in a schematic form in Fig. 1.

### Analysis

Though blinding of the patients is not possible for a behavioral intervention such as this, the assessment of the outcome, which is an objective measurement, will be done in a blinded fashion. All the study personnel collating and analyzing this data will be blinded to the treatment assignment. In addition, the laboratory personnel who will be measuring the urinary sodium (which is the primary outcome) will be blinded to treatment assignment.

We will have two primary efficacy outcomes, reduction in 24-hour urine sodium at four weeks and reduction in 24-hour urine sodium at 12 months. While we anticipate little or no loss to follow-up at four weeks, it is possible that there might be as much as a 30 % loss at 12 months. The minimal clinically important difference according to the CHEP guidelines is a 40 mmol reduction. Our review of the literature identified a range of possible standard deviations from 31 to 51 mmol. To be conservative, we assumed 51 mmol for the sample size estimation. Adjusting for multiple testing, this means that we would need 42 patients per group to have 90 % power in a t-test. In order to ensure this many patients at 12 months, we will recruit 60 patients per group. This calculation is conservative since it is based on a paired *t*-test but we will analyze the outcomes with the more powerful analysis of covariance (ANCOVA model.

Secondary outcomes will include changes in daytime average systolic BP by 24-hour ABPM, changes in dietary potassium intake assessed from 24-hour urinary potassium, and changes in body weight, all at the same time points as described for the primary outcome measure (four weeks as well as 12 months post-intervention). We will also do a one-page survey of participants at the end of the study (12 months) to identify potential barriers to salt restriction in diet.

All analysis will be conducted using the intention-to-treat principle. Any missing data will be imputed using multiple imputations. No interim analyses are planned, and there are no stopping rules for this trial. Subgroup analyses are planned based on tertiles of baseline sodium intake and source of high sodium intake (as identified by dietitian). We will also assess the association between changes in sodium, potassium intake, and changes in body weight and BP. In addition, we will conduct a sensitivity analysis based on achieved sodium intake and its relationship with BP. All the subgroup and sensitivity analyses will be hypothesis-generating and will be interpreted accordingly.

### Study management and patient safety

A trial management group involving the Principal Investigators (MR and SH), two Co-Investigators (TR, AK), and the Study Coordinator (JW) will review, implement, and supervise all aspects of this trial. Since this is a low risk intervention, consisting of counselling alone, we do not have a data safety and monitoring committee. Participants will be explicitly informed as part of the consent process that they can decide to withdraw from the study at any time if they so desire, without affecting their medical care.

### Ethical issues and trial registration

The trial will be conducted in accordance with Health Canada’s Good Clinical Practice guidelines and in accordance with the current Declaration of Helsinki and the Tri-Council Policy Statement: Ethical Conduct for Research Involving Humans [22, 23]. The study protocol and informed consent forms have been approved by the Ottawa Health Science Network Research Ethics Board (Protocol ID: 20140717-01H). The trial was registered at the US National Institutes of Health (Clinicaltrials.gov identifier: NCT02283697) on 2 November 2014.

## Discussion

High salt intake is directly responsible for elevated BP in a significant proportion of hypertensive patients. Reduction of salt consumption decreases BP in hypertensive patients. Low salt diets, such as the DASH-sodium, are fairly well established. However, counselling methods leading to reduced salt intake suitable for outpatients using available healthcare resources and patients’ time are not [18, 21]. In addition, even counselling methods that did result in reduced salt intake by methods such as hospitalization of patients and food provision, or many hours of counselling from dietitians for outpatients combined with food provision and/or access to communal kitchens, have been reported to lose their efficacy by six months after the active counselling ceased [20]. Hence there a need for an effective counselling method on low sodium intake pragmatically using available healthcare resources that can lead to a successful and sustained lower sodium intake.

Our approach took inspiration from a proven strategy of a one-time intensive counselling session followed by weekly telephone reminders which significantly improves adherence to BP lowering drug treatments [24, 25]. This new but proven method is adapted for counselling on low salt intake as we believe that if it is successful, it would be suitable for outpatient programs such as hypertension clinics or interprofessional family practices (family health teams). This study will also assess the long term efficacy of our counselling strategy, as well as more important outcome with regards to BP control population-wide. A negative, or partially negative (positive effect at four weeks with attrition by 12 months) trial outcome also has significant implications for healthcare delivery and use of resources. A completely negative trial should lead to a search for new methods to change patients’ most intimate behaviors such as dietary habits. A partially negative trial may just indicate that further interventions are necessary to help with persistence for some or all patients.

## Trial status

Trial recruitment is anticipated to begin in June 2015. The responsibility for study design, data collection, interpretation, and writing of the report and decision to submit the report for publication resides with the research team, and not the funding body or sponsor. The research team will have access and control over the trial dataset. The findings of the trial will be disseminated using presentation at national conferences (Canadian Hypertension Conference, Canadian Society of Nephrology) and by publication in peer-reviewed journals. We will follow the Consolidated Standards of Reporting Trials (CONSORT) checklist, along with the extensions for pragmatic trials [26] and non-pharmacologic interventions [27], in preparing the final report. The International Committee of Medical Journal Editors criteria will be used to determine authorship of the final trial results, with no usage of any professional writers. Trial participant-level data and statistical codes will be made available on request.

## Additional file

Additional file 1:
**SPIRIT 2013 Checklist.** Contains the recommended items to address in a clinical trial protocol and related documents based on Standard Protocol Items: recommendations for Interventional Trials (SPIRIT).

## Abbreviations

ABPM: Ambulatory blood pressure monitoring
BP: Blood pressure
CHEP: Canadian Hypertension Education Programme
DASH: Dietary approaches to stop hypertension
eGFR: Estimated glomerular filtration rate
